# Neural Field Theory of Evoked Response Potentials With Attentional Gain Dynamics

**DOI:** 10.3389/fnhum.2020.00293

**Published:** 2020-08-07

**Authors:** Tara Babaie-Janvier, Peter A. Robinson

**Affiliations:** ^1^School of Physics, University of Sydney, Sydney, NSW, Australia; ^2^Center of Excellence for Integrative Brain Function, University of Sydney, Sydney, NSW, Australia

**Keywords:** corticothalamic system, neural field theory, evoked potentials, brain filters, synaptic gain adjustment, local feedback, attention, brain oscillations

## Abstract

A generalized neural field model of large-scale activity in the corticothalamic system is used to predict standard evoked potentials. This model embodies local feedbacks that modulate the gains of neural activity as part of the response to incoming stimuli and thus enables both activity changes and effective connectivity changes to be calculated as parts of a generalized evoked response, and their relative contributions to be determined. The results show that incorporation of gain modulations enables a compact and physically justifiable description of the differences in gain between background-EEG and standard-ERP conditions, with the latter able to be initiated from the background state, rather than requiring distinct parameters as in earlier work. In particular, top-down gains are found to be reduced during an ERP, consistent with recent theoretical suggestions that the role of internal models is diminished in favor of external inputs when the latter change suddenly. The static-gain and modulated-gain system transfer functions are analyzed via control theory in terms of system resonances that were recently shown to implement data filtering whose gain adjustments can be interpreted as attention. These filters are shown to govern early and late features in standard evoked responses and their gain parameters are shown to be dynamically adjusted in a way that implements a form of attention. The results show that dynamically modulated resonant filters responsible for the low-frequency oscillations in an evoked potential response have different parameters than those responsible for low-frequency resting EEG responses, while both responses share similar mid- and high-frequency resonant filters. These results provide a biophysical mechanism by which oscillatory activity in the theta, alpha, and beta frequency ranges of an evoked response are modulated as reflections of attention; notably theta is enhanced and alpha suppressed during the latter parts of the ERP. Furthermore, the model enables the part of the ERP response induced by gain modulations to be estimated and interpreted in terms of attention.

## 1. Introduction

Evoked related potentials (ERPs) are scalp voltage responses to modulated stimuli. These are widely used to probe sensory processing and its technique has proved particularly valuable for testing theories of perception and attention (Picton et al., 2000; Herrmann and Knight, 2001; Kotchoubey, 2005; Womelsdorf et al., 2007; Woodman, 2010). When the visual stimulus is periodic with a frequency of at least 4 Hz the response overlaps into a quasisinusoidal oscillation to form a steady state visual evoked potential (SSVEP) with frequency and phase locked to the stimulus. The early part of the ERP response to visual stimuli is correlated with selective attention, processing of color, shape, and rotation (Kotchoubey, 2005), whereas later parts of the response are related to discrimination tasks, revealing more complex cognitive processes (Kotchoubey, 2005).

An ERP characteristic called visual mismatch negativity (MMN) is the difference between the “standard” response to a frequent stimulus and the “deviant” or “target” response to an infrequent stimulus that is interleaved with the frequent one. This is the visual analog of the original auditory mismatch negativity (Näätänen et al., 1978; for a review see Näätänen et al., 2010). This relatively simple electrophysiological measure is widely used to assess cognitive decline and hence functional deficiency associated with aging and many neuropsychiatric and neurological disorders (Kujala et al., 2007; Winkler, 2007; Näätänen et al., 2011).

**Figure 1:**
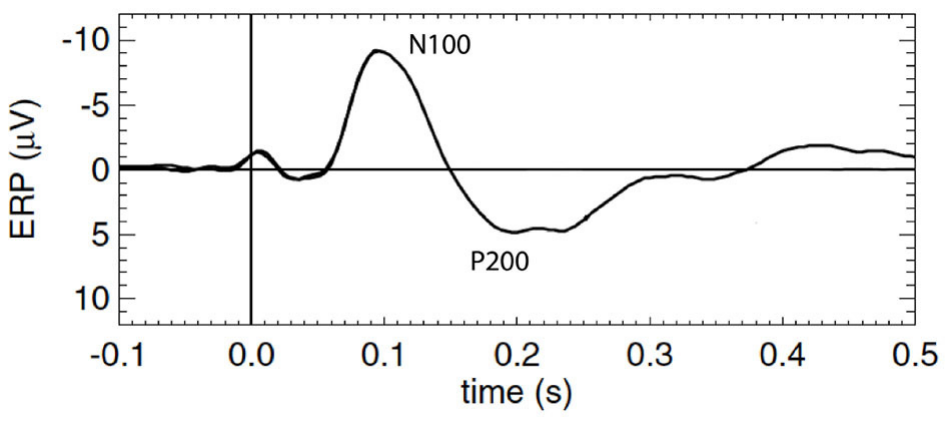
Schematic of a typical standard cortical ERP with prominent early and late components labeled N100 and P200, respectively.

To better understand ERPs and MMN, one must first understand the mechanism by which the background or standard evoked potential is generated, which is the focus of this study. The waveforms are traditionally described in terms of phenomenological ERP “components,” each of which is a peak or trough that is distinguished by its timing, polarity, and magnitude, with all other points in the ERP waveform being discarded; sometimes scalp distribution and sensitivity to task manipulations are also considered (Picton et al., 2000; Handy, 2005; Kotchoubey, 2005). ERP dynamics have been widely studied and associated with early and late information processing; however, relatively few studies have linked them explicitly to underlying mechanisms. In the present work we focus on the standard ERP that results from a repetitively applied stimulus that contains no novelty and to which subjects pay no conscious attention (Herrmann, 2001; Kotchoubey, 2005), particularly the first few hundred milliseconds after the stimulus, which contains the so-called N100 and P200 features, N100 being a negative deflection at around 100 − 150 ms post-stimulus, and P200 being a positive deflection at around 200 − 500 ms. Figure 1 shows a schematic of a typical background cortical ERP. The early components, peaking in roughly the first 100 ms, are termed “sensory” or “exogenous” because they depend largely on the physical parameters of the stimulus (Peterson et al., 1995; Handy, 2005; Lakatos et al., 2007), while the later responses reflect internal processing and are termed “cognitive” or “endogenous” (Handy, 2005). In what follows we use N100 and P200 as convenient labels for the relevant features, but completely avoid analysis in terms of “components” in order to treat all the time series data on an equal footing, instead of focusing on isolated points.

Physiological changes underlie the observed electrical response, so ERPs contain information about how the brain carries out stimulus processing (Kotchoubey, 2005). It has been shown that ERPs display systematic changes in amplitude and timing depending on the stimulus characteristics, task instructions, age, and neurological disorders (Picton et al., 2000; Womelsdorf et al., 2007; Kerr et al., 2008, 2011; Woodman, 2010). Hence, the modulation of physiological parameters that leads to these conditions results in consistent, reproducible changes in the ERP. To extract maximum information from observed ERPs requires them to be related to the underlying physiology, potentially allowing physiology and function to be inferred from ERP features, rather than their analysis being limited to cataloging peaks and troughs and finding correlations. Despite many proposals regarding the physiological processes involved in ERPs, the basic mechanisms of such modulation are, nonetheless, poorly understood.

ERP techniques have been widely used to unveil key aspects of attention which are unobservable with conventional behavioral techniques. Attention permits us to focus on certain aspects of upcoming information at the expense of less relevant ones. EEG-correlates of attention measured by ERPs include changes in the amplitude and timing of salient features. Most notably, both early and later features of ERPs are significantly enhanced in attentionally modulated responses (Hillyard and Anllo-Vento, 1998; Hillyard et al., 1998; Herrmann and Knight, 2001). Brain oscillations are also among correlates of attention which have been only studied recently, including modulation of alpha (≈7.5 − 12 Hz) and beta (≈12.5 − 30 Hz) oscillations due to attention (Herrmann and Knight, 2001; Marrufo et al., 2001; Ward, 2003; Yamagishi et al., 2003; Sauseng et al., 2005; Thut et al., 2006). However, the temporal dynamics of such activity modulation and its relationship to stimuli are not yet established.

In recent decades many brain phenomena have been explained in terms of physiologically based neural field theory (NFT) of the corticothalamic system in which microscopic structure is averaged over (Robinson et al., 1997, 2002, 2004; Rennie et al., 2002; Robinson and Roy, 2015). The NFT equations are nonlinear in general and have been successfully employed to explain both linear dynamics and highly nonlinear phenomena such as epileptic seizures (Robinson et al., 2002; Breakspear et al., 2006). Steady states of corticothalamic NFT have been shown to underlie normal brain dynamics, with small linear perturbations representing time-dependent brain activity (Robinson et al., 1997, 1998, 2002, 2004). This approximation has enabled a large variety of experimental phenomena, in cohorts of up to 2,100 subjects, to be reproduced, including ERPs and steady-state evoked responses (O'Connor and Robinson, 2004; Rowe et al., 2004; Robinson et al., 2005; Kerr et al., 2008; van Albada et al., 2010; Roberts and Robinson, 2012; Abeysuriya et al., 2015). Certainly, linear responses must be thoroughly understood before proceeding to nonlinear cases.

NFT of the corticothalamic system has also been used to study ERPs (Rennie et al., 2002; Kerr et al., 2008). In this case, the model was used in its original form where the neural gains were fixed throughout the process; we refer to this as a static-gain system. These authors found that the starting point of ERPs had rather different parameters than those that applied for background EEG, whereas one might have thought that they should be the same prior to arrival of the stimulus. This raises several key questions regarding the connectivity parameters: Why should we need a whole extra set of parameters to explain ERPs? How does attention affect ERP waveforms and through what mechanism? How does the brain adjust its connectivity (i.e., gains) on short timescales during an evoked response. Here we argue that, instead of postulating a new set of baseline parameters, the gains are modulated by known mechanisms as part of the evoked response itself—including habituation, facilitation, and similarly well-established effects (Koch, 1999; Rennie et al., 1999, 2002). In particular, we examine whether these feedbacks can account for attentional modulation in response to new information (Babaie-Janvier and Robinson, 2019).

The present work uses the generalized NFT of the corticothalamic system developed by Babaie-Janvier and Robinson (2018) in which feedback loops change gains in response to stimuli, driven by changes in presynaptic and/or postsynaptic activity caused by local feedbacks that directly affect the synaptic strength (Koch, 1999; Rennie et al., 1999, 2002; Robinson and Roy, 2015), which we refer to as the dynamically-modulated system. These local feedbacks are formulated in a sufficiently general way to describe a broad range of specific biophysical mechanisms such as plasticity, long-term potentiation/depression, facilitation, habituation, and sensitization (Rennie et al., 1999, 2002; Robinson and Roy, 2015). This yields a tractable representation of dynamic gain modulations that are part of the system's linear stimulus response. Of course, if the modulations were to become sufficiently large, a fully nonlinear analysis would be necessary. Our model is then used to analyze the stimulus-driven cortical response in terms of low-frequency, alpha, and beta resonances that can be interpreted as implementing standard data filters that predict the input and attend to their changes (Babaie-Janvier and Robinson, 2018, 2019).

Our aims are: (i) to predict the standard ERP when the driving signal is not so strong as to induce nonlinear features such as entrainment, harmonics, or subharmonics, which are left for future work, (ii) to study the role of gain modulations in time-dependent features of ERP and their relationship to attention, (iii) to determine the part of ERP response that is due to gain adjustments and whether these can be interpreted as implementing attention, (iv) to determine the corticothalamic filters that govern oscillatory correlates of attention in ERPs and their relationship with attention, and (v) to highlight general aspects of the results that are likely to be valid beyond the specific model studied here. We focus on three central cases: the ERP that would be evoked starting from the static gains that produce background EEG activity in the model; the ERP that is produced by choosing a separate set of static parameters to get a good match with observed standard ERP waveforms; and the ERP produced by starting from background EEG parameters, but allowing them to evolve dynamically as part of the response.

Section 2 briefly reviews the fundamental of the physiology based, dynamic-gain NFT model of the corticothalamic system. In section 3, we employ the model to produce ERPs and relevant comparisons with the current literature are presented. In section 4, we conclude and discuss future directions.

## 2. Materials and Methods

Here we summarize the relevant aspects of the use of physiologically based neural field theory in modeling large scale brain activity and outline the essential components of corticothalamic system. Full details can be found in Babaie-Janvier and Robinson (2018, 2019) and Robinson et al. (2002, 2004).

### 2.1. NFT of Corticothalamic System

Our model employs physiologically based neural field theory that permits tractable prediction and analysis from the microscale to the whole brain. Numerous experimental outcomes in normal and abnormal states, ranging from spontaneous activity to stimulus responses, evolution of sleep-wake cycles, neural plasticity, and epileptic seizures have been successfully reproduced using this model, as noted in section 1.

Our model, shown in Figure 1A, incorporates the cortex and thalamus and their connectivities; each includes distinct population of neurons: cortical excitatory (*e*) and inhibitory (*i*) neurons, the thalamic reticular nucleus (TRN) (*r*), thalamic relay neurons (*s*), and noncorticothalamic neurons that provide external inputs (*n*). In this study the relevant relay nucleus is the lateral geniculate nucleus (LGN), whose projections are to primary visual cortex (V1). The model incorporates the visual projection system with reciprocal corticothalamic feedback projections, excitatory projections to the TRN from LGN-V1 feedforward axons and from V1-LGN feedback axons, and inhibitory projections from the TRN onto LGN relay neurons.

The state of each neural population *a*, is represented by the local mean cell-body potential *V*_*a*_, the mean rate of firing at the cell body *Q*_*a*_, and the propagating axonal pulse rate field ϕ_*a*_. NFT averages over short spatial and temporal scales larger than a few tenths of a millimeter to obtain equations for the evolution of these dynamical variables (Wilson and Cowan, 1973; Freeman, 1975; Deco et al., 2008).

The mean firing rates *Q*_*a*_ exhibit a sigmoidal response to increasing mean soma voltage *V*_*a*_ measured relative to resting, which can be approximated by (Wilson and Cowan, 1973; Freeman, 1975; Deco et al., 2008)

(1) $$\begin{array}{l} Q_{a}\left(\text{r},t\right)=S\left(V_{a}\right)=\frac{Q_{\text{max}}}{1+\exp\left\{-\left[V_{a}\left(\text{r},t\right)-\theta \right]/\sigma^{\prime }\right\}}, \end{array}$$

where θ is the mean neural firing threshold and $\sigma^{\prime }\pi /\sqrt{3}$ is the standard deviation of the difference between the steady state soma voltage of individual neurons and their thresholds.

The net effect *V*_*a*_(**r**, *t*) on the activity of neurons of population *a* by all afferent neural synaptic receptors of type *b* is given by and

(2) $$\begin{array}{l} D_{\alpha }\left(t\right)V_{a}\left(\text{r},t\right)=\underset{b}{\sum }N_{ab}s_{ab}\phi_{b}\left(\text{r},t-\tau_{ab}\right), \end{array}$$

(3) $$\begin{array}{l} D_{\alpha }\left(t\right)=\frac{1}{\alpha \beta }\frac{d^{2}}{dt^{2}}+\left(\frac{1}{\alpha }+\frac{1}{\beta }\right)\frac{d}{dt}+1, \end{array}$$

where the differential operator *D*_α_ governs the temporal response of *V*_*ab*_ to afferent pulse rate fields ϕ_*b*_ encapsulating the rates β and α of the rise and fall, respectively, of the response at the cell body, *N*_*ab*_ is the mean number of synapses on neurons *a* from neurons of type *b*, *s*_*ab*_ is the mean time-integrated strength of soma response per incoming spike, and ϕ_*b*_(**r**, *t* − τ_*ab*_) is the mean spike arrival rate from neurons *b*, delayed by τ_*ab*_ due to discrete anatomical separations between different populations. The overall connection strength between two neural populations of types *a* and *b* is ν_*ab*_ = *N*_*ab*_*s*_*ab*_. In our model ν_*ie*_ = ν_*ee*_, ν_*ii*_ = ν_*ei*_, and ν_*is*_ = ν_*es*_ because the number of cortical synapses is closely proportional to the numbers of source and target neurons (Wright and Liley, 1996; Robinson et al., 1997; Braitenberg and Schüz, 1998), assuming the strength of synapses is determined by the source neurons. Forward time delays are τ_*es*_ = τ_*is*_ ≈ 20 ms corresponding to thalamocortical propagation and backward delays are τ_*se*_ = τ_*re*_ ≈ 60 ms, which correspond to corticothalamic propagation, while the remainder of the τ_*ab*_ are zero.

In our model, neural pulses within each population are averaged over short scales to form a field ϕ_*a*_(**r**, *t*) whose source is *Q*_*a*_(**r**, *t*), that propagates at a velocity *v*_*a*_, and which approximately obeys the spatiotemporal damped wave equation $D_{a}\left(\text{r},t\right)$ (Jirsa and Haken, 1996; Robinson et al., 1997),

(4) $$\begin{array}{l} D_{a}\left(\text{r},t\right)\phi_{a}\left(\text{r},t\right)=Q_{a}\left(\text{r},t\right), \end{array}$$

(5) $$\begin{array}{l} D_{a}\left(\text{r},t\right)=\frac{1}{\gamma_{a}^{2}}\frac{\partial^{2}}{\partial t^{2}}+\frac{2}{\gamma_{a}}\frac{\partial }{\partial t}+1-r_{a}^{2}\nabla^{2}. \end{array}$$

Here the damping rate γ_*a*_ satisfies γ_*a*_ = *v*_*a*_/*r*_*a*_, where *r*_*a*_ and *v*_*a*_ are the characteristic range and conduction velocity of axons of type *a*. In the corticothalamic system, only the axons of excitatory cortical neurons are long enough to cause propagation effects, which are included via Equation (5). In the other populations, we assume the axonal length to be small enough that it can be neglected (i.e., *r*_*a*_ ≈ 0, so $D_{a}\approx 1$), which results in ϕ_*a*_(**r**, *t*) = *Q*_*a*_(**r**, *t*) for these populations.

Table 1 lists nominal values of model parameters (Robinson et al., 2004) for resting EEG. These values were estimated for normal adults and they have been extensively used and verified in comparisons with experiments, as mentioned in section 1.

**Table 1:** Estimated brain parameters for normal adults in the alert eyes-open state.

**Quantity**	**Description**		**Resting**	**Standard ERP**	
			**EEG**	**Kerr et al**.	**Unit**
*Q*_max_	Max firing rate		250	250	s^−1^
θ	Firing threshold		15	15	mV
σ′	Threshold spread		3.3	3.3	mV
γ_*e*_	Cortical damping rate		116	200	s^−1^
α_*ab*_	Inverse decay time		80	45	s^−1^
β_*ab*_	Inverse rise time		320	180	s^−1^
τ_*es*_	Forward delay time		20	32	ms
τ_*se*_	Feedback delay time		60	32	ms
**Firing rate**					
$\phi_{e}^{\left(0\right)}$	Steady-state firing rate of *e* neurons		16	16	s^−1^
$\phi_{s}^{\left(0\right)}$	Steady-state firing rate of *s* neurons		16	16	s^−1^
$\phi_{r}^{\left(0\right)}$	Steady-state firing rate of *r* neurons		16	16	s^−1^
$\phi_{n}^{\left(0\right)}$	Steady-state firing rate of *n* neurons		16	16	s^−1^
**Sigmoid slope**					
ρ_*e*_	for *e* Neurons		4.2 × 10^3^	4.2 × 10^3^	V^−1^ s^−1^
ρ_*s*_	for *s* Neurons		4.2 × 10^3^	4.2 × 10^3^	V^−1^ s^−1^
ρ_*r*_	for *r* Neurons		6.3 × 10^3^	6.3 × 10^3^	V^−1^ s^−1^
**Synaptic gain (dimensionless)**					
$G_{ee}^{\left(0\right)}$	Steady-state gain to *e* from *e*		6.8	3.1	−
$G_{se}^{\left(0\right)}$	Steady-state gain to *s* from *e*		2.5	1.18	−
$G_{ii}^{\left(0\right)}$	Steady-state gain to *i* from *i*		−8.1	−10.8	−
$G_{sr}^{\left(0\right)}$	Steady-state gain to *s* from *r*		−1.9	−2.8	−
$G_{es}^{\left(0\right)}$	Steady-state gain to *e* from *s*		1.7	0.74	−
$G_{sn}^{\left(0\right)}$	Steady-state gain to *s* from *n*		0.8	0.8	−
$G_{ie}^{\left(0\right)}$	Steady-state gain to *i* from *e*		6.8	3.1	−
$G_{re}^{\left(0\right)}$	Steady-state gain to *r* from *e*		1.0	3.4	−
$G_{ei}^{\left(0\right)}$	Steady-state gain to *e* from *i*		−8.1	−10.8	−
$G_{rs}^{\left(0\right)}$	Steady-state gain to *r* from *s*		0.19	0.28	−
$G_{is}^{\left(0\right)}$	Steady-state gain to *i* from *s*		1.7	0.74	−
**Stability parameters (dimensionless)**					
*X*	Cortical stability		0.7	0.3	−
*Y*	Corticothalamic stability		0.2	−0.4	−
*Z*	Intrathalamic stability		0.1	0.1	−

*The first two columns give the symbol and description of each quantity. The third column shows resting-EEG values adapted from Table 1 of Robinson et al. (2004); these are also used as the initial values for ERPs when gains evolve dynamically. The fourth column lists static-gain ERP values adapted from Table 1 of Kerr et al. (2008) which were previously used to match ERPs without gain modulation. The final column gives the units*.

### 2.2. Corticothalamic Transfer Functions

The above NFT equations are nonlinear in general. By setting all derivatives in these equations to zero, we find spatially uniform steady states of the system, which are interpreted as characterizing the baseline of normal activity, with firing rates that are in accord with experiment (Robinson et al., 2002, 2004). Linear perturbations from these steady states have been shown to correspond to time dependent brain activity, leading to successful comparisons with numerous experimental phenomena, including evoked responses (Robinson et al., 1997, 2002, 2004, 2005; Rennie et al., 2002; O'Connor and Robinson, 2004; Kerr et al., 2008; van Albada et al., 2010; Roberts and Robinson, 2012; Abeysuriya et al., 2015).

#### 2.2.1. Perturbation Expansion

We expand the equations in section 2.1 to first order in perturbations relative to the steady state, denoting steady-state and perturbed quantities by the superscripts 0 and 1, respectively. We then find

(6) $$\begin{array}{l} Q_{a}^{\left(0\right)}+Q_{a}^{\left(1\right)}\left(\text{r},t\right)=S\left[V_{a}^{\left(0\right)}\right]+\rho_{a}V_{a}^{\left(1\right)}, \end{array}$$

(7) $$\begin{array}{l} D_{\alpha }\left(t\right)\left[V_{a}^{\left(0\right)}+V_{a}^{\left(1\right)}\left(\text{r},t\right)\right]=\underset{b}{\sum }\left[\nu_{ab}^{\left(0\right)}+\nu_{ab}^{\left(1\right)}\left(\text{r},t\right)\right]\\ \;\left[\phi_{b}^{\left(0\right)}+\phi_{b}^{\left(1\right)}\left(\text{r},t-\tau_{ab}\right)\right], \end{array}$$

(8) $$\begin{array}{l} D_{a}\left(\text{r},t\right)\left[\phi_{a}^{\left(0\right)}+\phi_{a}^{\left(1\right)}\left(\text{r},t\right)\right]=Q_{a}^{\left(0\right)}+Q_{a}^{\left(1\right)}\left(\text{r},t\right), \end{array}$$

(9) $$\begin{array}{l} \rho_{a}=\frac{dQ_{a}}{dV_{a}}{|}_{V_{a}=V_{a}^{\left(0\right)}}. \end{array}$$

To zeroth order, Equations (6)–(8) yield

(10) $$\begin{array}{l} Q_{a}^{\left(0\right)}=S\left[V_{a}^{\left(0\right)}\right], \end{array}$$

(11) $$\begin{array}{l} V_{a}^{\left(0\right)}=\underset{b}{\sum }\nu_{ab}^{\left(0\right)}\phi_{b}^{\left(0\right)}, \end{array}$$

(12) $$\begin{array}{l} \phi_{a}^{\left(0\right)}=Q_{a}^{\left(0\right)}. \end{array}$$

Equations (10) and (12) can then be used to eliminate the other variables in favor of the $V_{a}^{\left(0\right)}$, which yields the nonlinear steady-state equation (Robinson et al., 1998, 2004)

(13) $$\begin{array}{l} V_{a}^{\left(0\right)}=\underset{b}{\sum }\nu_{ab}^{\left(0\right)}S\left[V_{b}^{\left(0\right)}\right]. \end{array}$$

The first order terms in Equations (6)–(8) give

(14) $$\begin{array}{l} Q_{a}^{\left(1\right)}\left(\text{r},t\right)=\rho_{a}V_{a}^{\left(1\right)}, \end{array}$$

(15) $$\begin{array}{l} D_{\alpha }\left(t\right)V_{a}^{\left(1\right)}\left(\text{r},t\right)=\underset{b}{\sum }\left[\nu_{ab}^{\left(0\right)}\phi_{b}^{\left(1\right)}\left(\text{r},t-\tau_{ab}\right)+\nu_{ab}^{\left(1\right)}\left(\text{r},t\right)\phi_{b}^{\left(0\right)}\right], \end{array}$$

(16) $$D_{a}(r,t)\phi_{a}^{\left(1\right)}(r,t)]=Q_{a}^{\left(1\right)}(r,t),$$

Operation with *D*_α_ on both sides of Equation (16), plus use of Equation (14), yields

(17) $$\begin{array}{l} D_{\alpha }\left(t\right)D_{a}\left(\text{r},t\right)\left[\phi_{a}^{\left(1\right)}\left(\text{r},t\right)\right]=\rho_{a}D_{\alpha }\left(t\right)V_{a}^{\left(1\right)}\left(\text{r},t\right), \end{array}$$

(18) $$\begin{array}{l} =\underset{b}{\sum }\left[G_{ab}^{\left(0\right)}\phi_{b}^{\left(1\right)}\left(\text{r},t-\tau_{ab}\right)+G_{ab}^{\left(1\right)}\left(\text{r},t\right)\phi_{b}^{\left(0\right)}\right], \end{array}$$

(19) $$\begin{array}{l} G_{ab}^{\left(0\right)}=\rho_{a}\nu_{ab}^{\left(0\right)}=\rho_{a}N_{ab}s_{ab}^{\left(0\right)}, \end{array}$$

(20) $$\begin{array}{l} G_{ab}^{\left(1\right)}\left(\text{r},t\right)=\rho_{a}\nu_{ab}^{\left(1\right)}\left(\text{r},t\right)=\rho_{a}N_{ab}s_{ab}^{\left(1\right)}\left(\text{r},t\right). \end{array}$$

The gain *G*_*ab*_(**r**, *t*) is the differential response in ϕ_*a*_ per unit change in incoming ϕ_*b*_. The net gains of populations of neurons connected serially are denoted by *G*_*abc*_ = *G*_*ab*_*G*_*bc*_ and *G*_*abcd*_ = *G*_*ab*_*G*_*bc*_*G*_*cd*_. These gains are parameterized by time, as shown in Equation (20) to represent their dynamics, which is the topic of the next section.

#### 2.2.2. Modulation of Synaptic Gains

Numerous biophysical processes modulate neuronal coupling strengths, dependent on current or recent activity, including plasticity, long-term potentiation/depression, facilitation, habituation, and sensitization. We employ a general form of modulatory process that can be applied to a broad range of specific mechanisms (Koch, 1999; Rennie et al., 1999; Robinson et al., 2002), which is a form of feedback, whereby presynaptic neuronal activity modulates neuronal gains (postsynaptic involvement is omitted here and postponed to future work), with

(21) $$\begin{array}{l} G_{ab}^{\left(1\right)}\left(\text{r},t\right)=g_{ab}F\left(t\right)⊗\phi_{b}^{\left(1\right)}\left(\text{r},t\right), \end{array}$$

where *F*(*t*) describes the temporal dynamics of the gain modulation and *g*_*ab*_ is its strength and ⊗ denotes convolution operation. Equation (21) assumes that the perturbations are small enough that a linear equation is a reasonable approximation. Furthermore, the modulation is assumed to be local in space, so the *g*_*ab*_ are constant and the functional form of *F*(*t*) does not vary with position or time. For the temporal function of the modulation we use (Robinson et al., 2004)

(22) $$\begin{array}{l} F\left(t\right)=\eta \exp\left(-\eta t\right), \end{array}$$

when *t* ≥ 0 and zero otherwise to enforce causality. The rate constant η > 0 characterizes the timescale of the feedback process.

#### 2.2.3. Transfer Functions

The transfer function is the ratio of the output of a system to its input in the linear regime. Either the Laplace or Fourier transform can be used to determine transfer functions; we use the former in time and the latter in space, with the definitions

(23) $$\begin{array}{l} L\left[f\left(t\right)\right]\left(s\right)=f\left(s\right)=\int_{0}^{\infty }f\left(t\right)e^{-st}dt, \end{array}$$

(24) $$\begin{array}{l} F\left[f\left(t\right)\right]\left(\omega \right)=L\left[f\left(t\right)\right]{|}_{s=-i\omega }, \end{array}$$

(25) $$\begin{array}{l} =\int_{-\infty }^{\infty }f\left(t\right)e^{i\omega t}dt, \end{array}$$

respectively, where *s* = −*iω* = Γ − iΩ is the complex frequency that parameterizes the response *e*^*st*^.

Replacement of $G_{ab}^{\left(1\right)}$ in Equation (18) by Equation (21) yields

(26) $$\begin{array}{l} D_{\alpha }(t)D_{a}(r,t)\left[\phi_{a}^{\left(1\right)}\left(k,s\right)\right]=\sum_{b}[G_{ab}^{\left(0\right)}\phi_{b}^{\left(1\right)}(r,t-\tau_{ab})\\ \;+g_{ab}F(t)⊗\phi_{b}^{\left(1\right)}(r,t)\phi_{b}^{\left(0\right)}]. \end{array}$$

Application of (23) and (24) to Equation (26) gives

(27) $$\begin{array}{l} D_{a}(k,s)\phi_{a}^{\left(1\right)}\left(k,s\right)=L(s)\sum_{b}[G_{ab}^{\left(0\right)}\exp(-s\tau_{ab})\\ \;\;+g_{ab}\phi_{b}^{\left(0\right)}F(s)]\phi_{b}^{\left(1\right)}(k,s), \end{array}$$

where *L*(*s*) is the reciprocal of the Laplace transform of the operator *D*_α_(*t*).

Equation (27) expresses first order responses of two types: the first term in the square brackets represents the part of response that would occur without change to the steady-state gains, whereas the second term is the response due to stimulus-induced gain changes of the steady-state activity. In the Laplace domain, the transfer function to excitatory cortical neurons from retina, is (see Babaie-Janvier and Robinson, 2018 for detailed derivation)

(28) $$\begin{array}{l} T_{en}\left(\text{k},s\right)=\frac{\phi_{e}^{\left(1\right)}\left(\text{k},s\right)}{\phi_{n}^{\left(1\right)}\left(\text{k},s\right)}, \end{array}$$

(29) $$\begin{array}{l} =\frac{G_{esn}L_{esn}}{M\left(1-G_{esrs}L_{esrs}\right)-G_{ese}L_{ese}+G_{esre}L_{esre}}, \end{array}$$

where $M=D_{ee}\left(1-G_{ei}L_{ii}\right)-G_{ee}L_{ee}$, *G*_*ab*_ = *G*_*ab*_(*s*) is the Laplace transform of *G*_*ab*_(*t*), and

(30) $$\begin{array}{l} G_{ab}\left(s\right)=G_{ab}^{\left(0\right)}\exp\left(-s\tau_{ab}\right)+g_{ab}\phi_{b}^{\left(0\right)}F\left(s\right). \end{array}$$

Note that Equation (29) corrects a typographical error in the corresponding equations in Babaie-Janvier and Robinson (2018); however, the paper used the correct equation and their results remain unchanged.

The transfer function fully describes the linear system properties including the linear response to any external signal. Poles of the transfer function yield the characteristic equation of the system, whose roots determine the poles and thus the basic modes into which the system response can be decomposed. Roots of the numerator of the transfer function are the zeros of the system; these frequencies do not pass through the system.

In this study we only explore spatially uniform perturbations (i.e., **k** = 0) and postpone study of spatial dependences to future work. Such spatially unstructured stimuli have been widely used in visual flicker experiments to probe steady state visual evoked potentials (SSVEPs) (Spekreijse et al., 1973; Herrmann, 2001; Roberts and Robinson, 2012; VanRullen and Macdonald, 2012). It was also shown recently that the spatially uniform contribution dominates the later phases of ERPs (Mukta et al., 2019).

### 2.3. Reduced Description in Terms of Loop Gains

**Figure 2:**
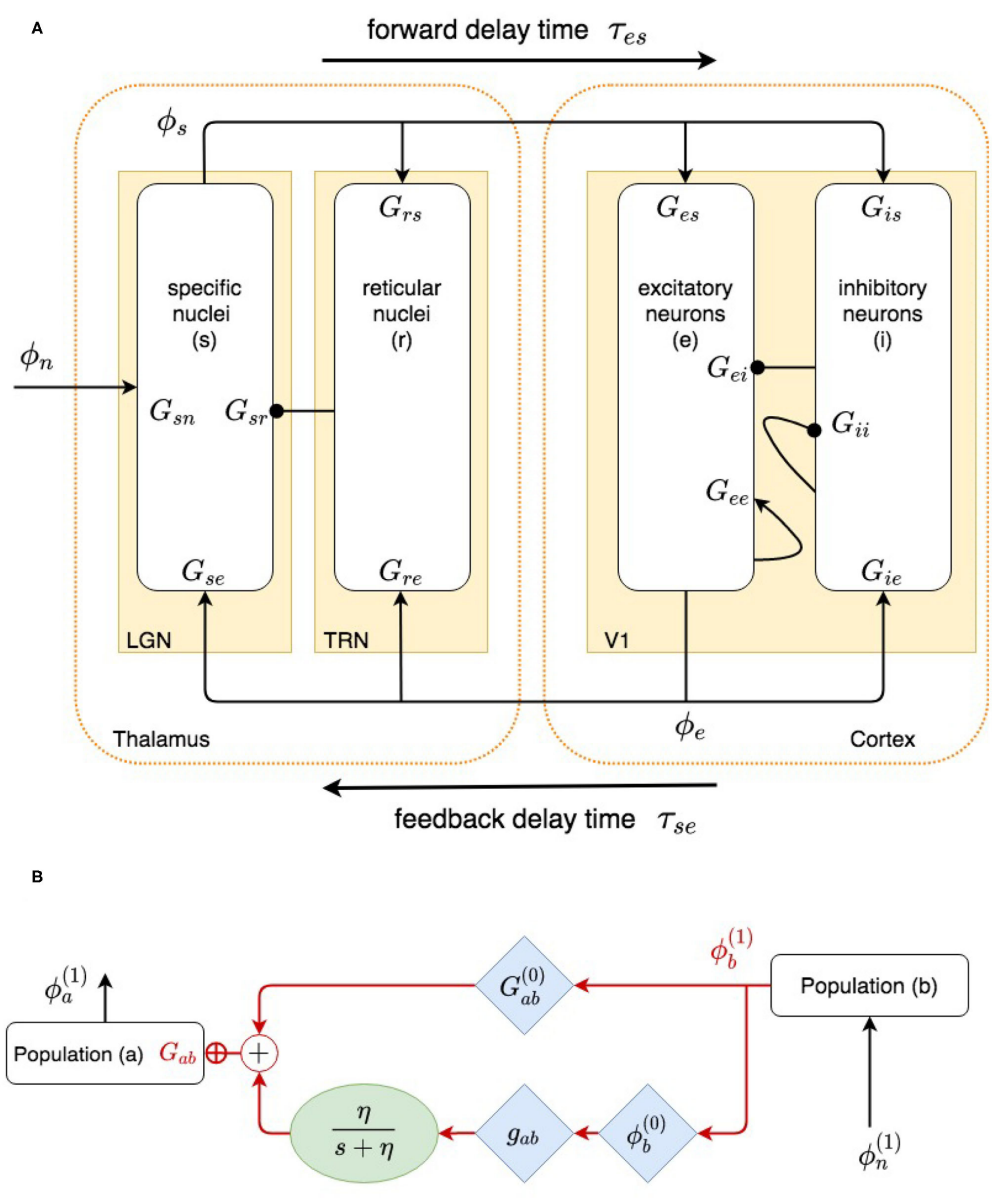
**(A)** Physiologically based corticothalamic model in which the arrows represent excitatory effects and the circles depict inhibitory ones. The populations are cortical excitatory (*e*) and inhibitory (*i*) neurons, the thalamic reticular nucleus (*r*), thalamic relay neurons (*s*) that project to the cortex, and non-corticothalamic neurons responsible for external inputs (*n*). **(B)** Schematic of dispersion of neural activity to population *a* from population *b*, where modulation of the neuronal gain by local feedback is given by Equation (21).

Previous work has shown that many aspects of corticothalamic dynamics can be approximately described in terms of normalized gains for the loops shown in Figure 2, and this simplified description is used below to assist interpretation. These gains are for (i) net cortical feedback directly on itself, denoted *X*; (ii) net feedback of the cortex on itself via the thalamus, denoted *Y*, including loops via just the relay nuclei and via both the reticular and relay nuclei, as shown in Figure 2; and (iii) feedback in the loop comprising the relay and reticular nuclei, denoted *Z* (Robinson et al., 2002; Abeysuriya et al., 2015; Robinson, 2017). These quantities are defined

(31) $$\begin{array}{l} X=\frac{G_{ee}}{1-G_{ei}}, \end{array}$$

(32) $$\begin{array}{l} Y=\frac{G_{ese}+G_{esre}}{\left(1-G_{ei}\right)\left(1-G_{srs}\right)}, \end{array}$$

(33) $$\begin{array}{l} Z=-G_{srs}\frac{\alpha \beta }{{\left(\alpha +\beta \right)}^{2}}. \end{array}$$

The requirement of brain stability confines steady state values of *X*, *Y*, and *Z* to a stability zone around the origin, beyond which seizures set in or the system moves to a different steady state (Robinson et al., 2002; Breakspear et al., 2006). However, instantaneous values outside this regime can be used to parameterize dynamics relative to steady states even when no instability exists. In approximate terms, large *X* implies that the cortex is mostly driven by its own activity and is thus introspective (Robinson, 2017). Large positive *Y* indicates strong feedback between cortex and thalamus and significant input from the external world (Robinson, 2017), whereas large negative *Y* is found in sleep and indicates high reticular activity with attendant suppression of relay nuclei and external inputs. The parameter *Z* is the strength of the intrathalamic loop formed by reciprocal connections between reticular and relay nuclei, which is responsible for sleep spindle generation but plays little role in the waking state (Robinson et al., 2002).

An important observation is that the dominant frequencies generated by the corticothalamic system tend to be near 0 Hz if *X* + *Y* ≈ 1, which is the brain's normal near-critical state (Robinson et al., 2002; Abeysuriya et al., 2015; Robinson, 2017). If, in addition, *Y* is large and positive and *Z* is not too near zero, an approximately 10 Hz alpha peak is generated along with its harmonic beta peak, both due to positive corticothalamic feedback. Values of *Y* near zero are usually associated with featureless spectra, while negative *Y* can lead to theta peaks via negative corticothalamic feedback. Large *Z* ≈ 1 is associated with spindle resonance in the intrathalamic loop. More detail and analysis of all these points can be found in the original references (Robinson et al., 2002; Abeysuriya et al., 2015).

### 2.4. Corticothalamic Data Filters

The transfer functions can be decomposed into basic modes whose behaviors are shown to be associated with data filters whose control system properties are well understood (Babaie-Janvier and Robinson, 2018). To do this we first note that each corticothalamic transfer function *T*_*ab*_(*s*) in Equation (28) is a ratio of exponential polynomials of *s*. If we approximate it by a rational function of *s* and decompose it into partial fractions, we find

(34) $$\begin{array}{l} T_{ab}\left(s\right)=\overset{n}{\underset{j\;=\;1}{\sum }}\frac{r_{j}}{s+p_{j}}; \end{array}$$

where the *p*_*j*_ = Γ_*j*_ ± iΩ_*j*_ are all distinct poles of the system (we do not consider the special case of degenerate roots here because this corresponds to a set of measure zero in parameter space), and the residues *r*_*j*_ = *r* ± iΩ_*r*_ are

(35) $$\begin{array}{l} r_{j}=\underset{s\to -p_{j}}{\lim}\left(s+p_{j}\right)T_{ab}\left(s\right), \end{array}$$

and *n* is the number of the poles, and also indicates the degree of the characteristic dispersion equation of the system. Some of these poles are associated with heavily damped modes and can be neglected; this has been shown to result in a 6-pole approximation (*n* = 6) that is accurate to within a root-mean-square (rms) fractional error of 0.02 for 0 to 150 Hz for the parameters in Table 1 (Babaie-Janvier and Robinson, 2018). These partial fractions then are summed in pairs that dominate in low (*f* ≲ 5 Hz), alpha (5 Hz ≲ *f* ≲ 15 Hz), and beta (15 Hz ≲ *f*) frequency regimes, respectively. We thus write

(36) $$\begin{array}{l} T_{bn}\left(s\right)\approx {\tilde{T}}_{bn}\left(s\right), \end{array}$$

(37) $$\begin{array}{l} =T_{bn}^{\ell }\left(s\right)+T_{bn}^{A}+T_{bn}^{B}\left(s\right), \end{array}$$

where *b* = *s, r, e* and $T_{bn}^{\ell }$ is the sum of the two poles in slow range, while $T_{bn}^{A}$ and $T_{bn}^{B}$ are the sums over the pairs of poles that represent oscillatory responses in the alpha and beta frequency ranges, respectively. The partial transfer function of the sum of two fractions associated with poles *p*_*j*_ and *p*_*j*+1_ either both real or a conjugate pair, which we denote by $T_{ab}^{ȷ}\left(s\right)$ for ȷ=ℓ,A,B, with

(38) $$\begin{array}{l} T_{ab}^{ȷ}\left(s\right)=\left(s+\tau_{p}^{-1}\right)\left[\frac{K}{\left(s+p_{j}\right)\left(s+p_{j+1}\right)}\right], \end{array}$$

with τ_*p*_ = (*r*_*j*_ + *r*_*j*+1_)/(*r*_*j*_*p*_*j*+1_ + *r*_*j*+1_*p*_*j*_) and *K* = *r*_*j*_ + *r*_*j*+1_. Each filter emphasizes the part of the signal from external world that lies within its frequency range; summing these parallel responses results in the total response.

## 3. Results

In this section, we employ the model described in section 2 to first examine the ERPs obtained using fixed nominal background-EEG parameters and the separate, also fixed, parameters used by Kerr et al. (2008) to fit experimental standard ERPs, both with static-gain transfer functions. We then turn on the gain modulation in the model, starting from background-EEG parameters as the initial prestimulus values and study the effects of gain dynamics on ERPs. To avoid confusion, we refer to the three cases and their parameters as background (which has static gains), static-gain ERP (Kerr et al.'s case), and modulated-gain ERP (the present generalization) below.

### 3.1. Evoked Potentials for Static-Gain Transfer Functions

**Figure 3:**
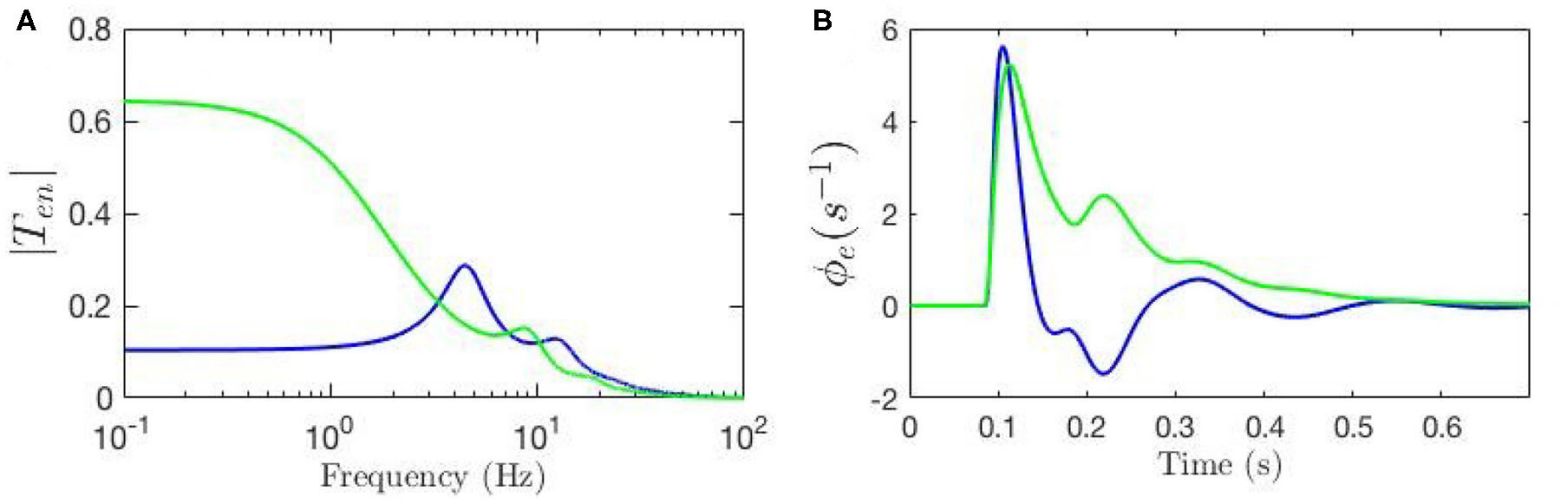
Comparison of the frequency and time responses calculated for nominal parameter values for eye-open normal adults in resting state (green line) and in ERP event (blue line) from Table 1. **(A)** Magnitude of the transfer functions. **(B)** Model cortical ERPs.

To evaluate the response $\phi_{e}^{\left(1\right)}$ of the cortical neurons to the stimulus $\phi_{n}^{\left(1\right)}$ using our model we must inverse Laplace transform the product of the transfer function and the stimulus signal; if the stimulus is a delta function this response corresponds to an ERP (Rennie et al., 1999; Kerr et al., 2008). Here, we fix all gains by setting the local synaptic strengths *g*_*ab*_ = 0 for *a, b* = *s, r, e, i*. We then calculate the ERP using nominal background values of model parameters and also reproduce the key result from Kerr et al. (2008) using their parameters; both sets of parameters are listed in Table 1. We denote the nominal background case by a circle superscript ($T_{en}^{\circ }$ and $\phi_{e}^{\circ }$), and the results from Kerr et al. (2008) by a star superscript ($T_{en}^{*}$ and $\phi_{e}^{*}$). Figure 3A shows these transfer functions and Figure 3B shows the corresponding ERP waveforms, which confirms that the differences in parameters significantly change the transfer function and the ERP.

Figure 3A shows that the two transfer functions feature different lower frequency peaks while they have similar high-frequency peaks. One observation is that alpha peak is present in both responses with lower magnitude although slightly different frequencies; the same observation applies for the beta peaks but these are weak. However, the key discrepancy between the two is present in the transfer functions' low-frequency regime where the ERP frequency response shows oscillations at ~ 4 Hz, but the resting-state transfer function does not. Figure 3B shows that the two ERPs also exhibit significant differences in terms of their prominent peaks and troughs (i.e., phenomenological “components”). The early N100 feature is present in both responses, whereas the later P200 feature is absent for the nominal resting values.

The key observation here is that when an ERP takes place, the characteristics of the system are shifted. One way to achieve such shifts during an ERP is through dynamic adjustment of the connectivities of the system so that the transfer function is altered from $T_{en}^{\circ }$ to $T_{en}^{*}$. In the other words, there can be underlying biophysiological mechanisms by which, during an ERP, gains are dynamically modulated so that the ERP waveform is modulated from $\phi_{e}^{\circ }$ to $\phi_{e}^{*}$. In our model, this can be achieved through local feedback modulation of gains as proposed in Equation (21). The key implication of using such a scheme for explaining the parameter adjustments during an ERP event is that these adjustments can be interpreted as implementing a form of attention (Babaie-Janvier and Robinson, 2018, 2019), which we discuss in detail in subsequent sections. The questions that arise of which changes impact the response, in which ways, and how strongly, are addressed in the next section.

### 3.2. Parameter Sensitivities

We observe that there is a discrepancy between the response generated by a nominal set of resting EEG parameters and the set used by Kerr et al. (2008) to fit to experimental ERPs. Our core aim here is to determine whether a comparably good fit can be obtained by instead starting from the resting-state gains but allowing them to be dynamically modulated as part of the response.

To investigate the root cause of the differences between the above two static-gain responses, and in order to guide our study of dynamic modulation of the gains, we first run a set of sensitivity analyses which help us determine the role of each parameter in the appearance and magnitude of the features of the response and transfer function. We use the set of parameters fitted for resting state EEG, and in each case, one parameter is varied while the rest are kept fixed. We first calculate transfer functions for the resting state using the parameters in Table 1. The parameter chosen for analysis is then changed to its value corresponding to Kerr's ERP parameter set and the new transfer function is calculated and plotted in blue. The parameter under investigation is then changed in the opposite direction to the one that yielded the values used by Kerr et al. (2008) and these are plotted in green.

**Figure 4:**
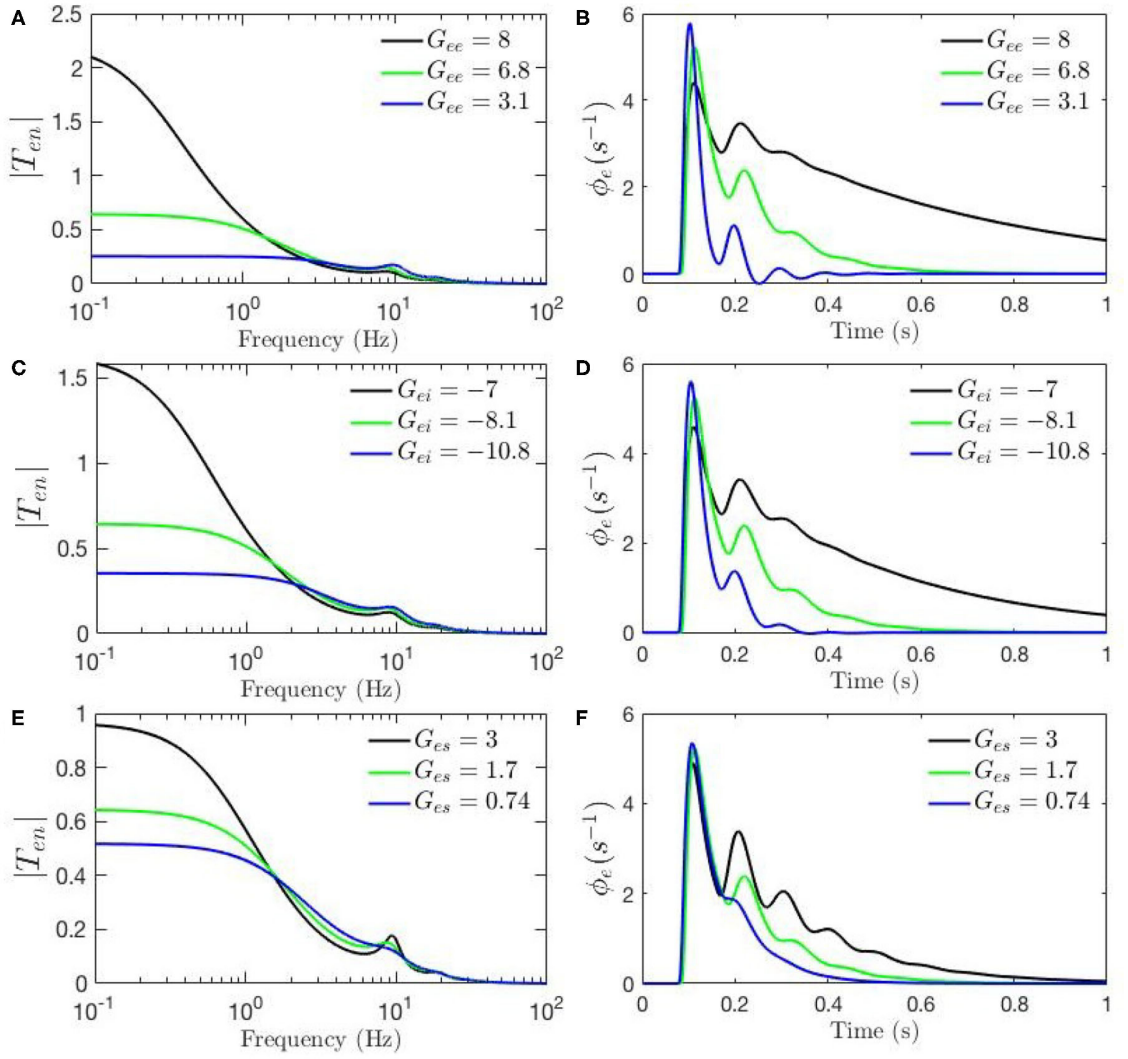
Sensitivity analyses of ERPs to cortical gains calculated using the transfer function corresponding to nominal parameter values for eyes-open normal adults in resting state EEG (black), as listed in Table 1. In each case one parameter is then changed to its value from the fixed-gain ERP set (blue), as also listed in Table 1, and then in the opposite direction relative to the EEG values (green). The parameters varied are as follows, with the transfer function *T* in the left column of each row and the corresponding ERP in the right column: **(A,B)** *G*_*ee*_. **(C,D)** *G*_*ei*_. **(E,F)** *G*_*es*_.

**Figure 5:**
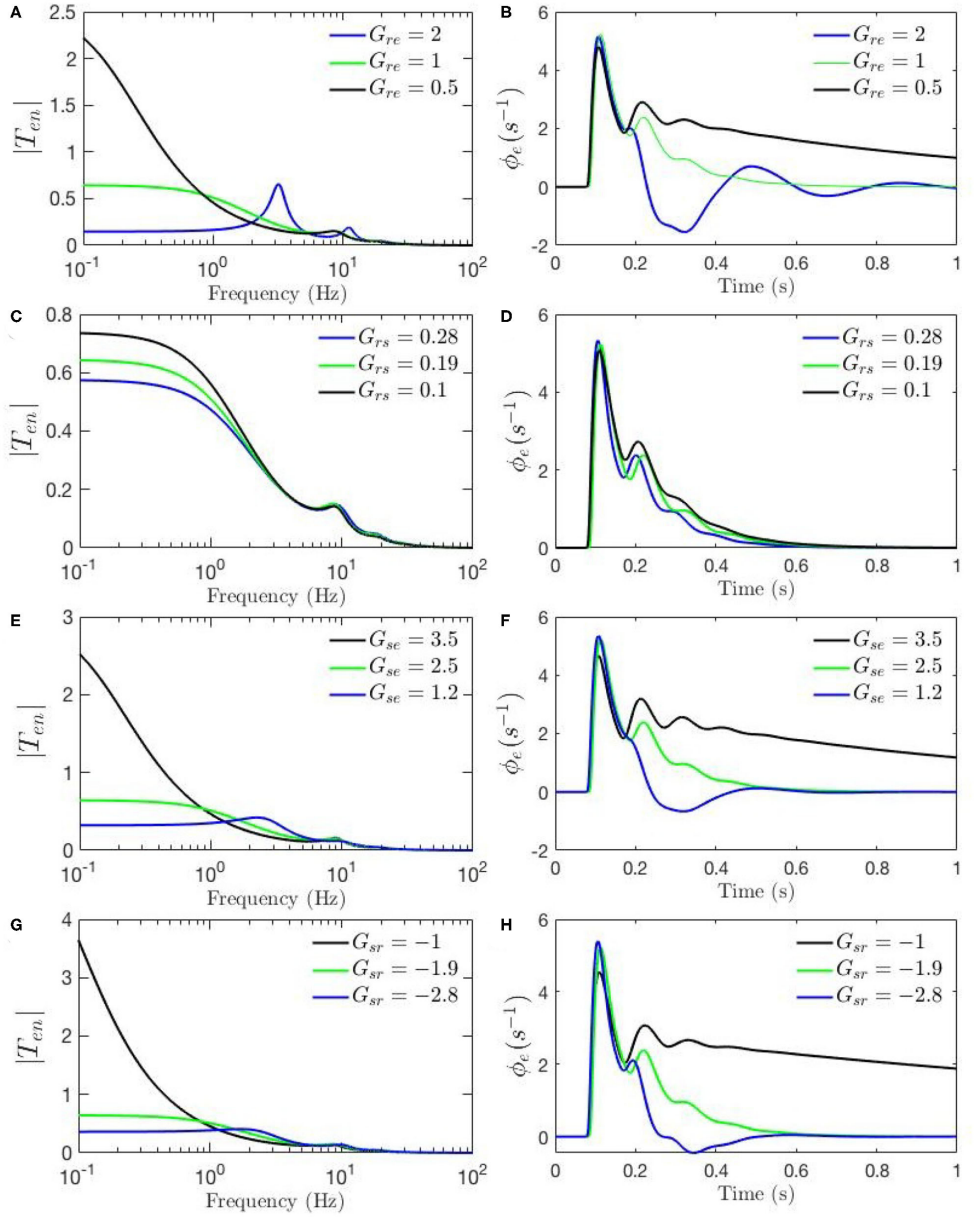
Sensitivity analyses of ERPs to corticothalamic and thalamic gains calculated using the transfer function corresponding to nominal parameter values for eyes-open normal adults in resting state EEG (black), as listed in Table 1. In each case one parameter is then changed to its value from the fixed-gain ERP set (blue), as also listed in Table 1, and then in the opposite direction relative to the EEG values (green). The parameters varied are as follows, with the transfer function *T* in the left column of each row and the corresponding ERP in the right column: **(A,B)** *G*_*re*_. **(C,D)** *G*_*se*_. **(E,F)** *G*_*rs*_. **(G,H)** *G*_*sr*_.

Figures 4A–F, 5A–H show ERPs obtained for the sensitivity analyses designed above. These results, in general, show that any change in each gain results in significant alteration of the shape of transfer functions and their corresponding ERPs. We observed in Figure 3A that the key difference between the two transfer functions is the presence of a theta peak (≈ 4 − 6 Hz) in the transfer function for ERP parameters. Figures 4A,C,E confirm that changes in cortical gains *G*_*ee*_, *G*_*ei*_, or *G*_*es*_, alone cannot induce such modifications in the transfer functions. On the other hand, Figures 5A,C,G show that such variations can be caused by modifications of the strength of synapses in the top-down pathways; i.e., *G*_*sr*_, *G*_*se*_, and *G*_*re*_. A decrease in both gains from cortex to TRN (*G*_*re*_ in Figure 5A) and gain from cortex to LGN (*G*_*se*_ in Figure 5C) will cause a theta peak to appear. A theta peak is also generated by a decrease in the magnitude of the inhibitory gain from TRN to LGN (*G*_*sr*_ in Figure 5G), also a top-down pathway. These observations suggest that changes in the top-down pathways play an important role during ERPs, in accord with the previously proposed hypothesis that upon occurrence of a sudden change (onset of stimulus) the brain attends to such information at the expense of less relevant top-down information (Friston, 2010; Babaie-Janvier and Robinson, 2019) which means during attention the role of internal model (top-down signals) is diminished while the data from outside brain is emphasized (Garrido et al., 2007; Friston, 2010, 2011; Clark, 2013).

The above observations allow us analyze the impact of the above changes on the normalized loop gains *X*, *Y*, and *Z*, and draw out the relationship between the oscillations and the stability parameters. *X*, which is constrained to positive values, is governed by *G*_*ee*_ and *G*_*ei*_. As Figures 4A–D show, alpha oscillations (and beta ones with lesser effect) are mildly modulated by decrease in *G*_*ee*_ and increase in *G*_*ei*_ which results in a decrease in *X*. A decrease in *X* indicates a significant decrease in the ratio of excitation to inhibition in the cortex. Theta resonance, in the other side, is not significantly modulated by cortical gains as seen in Figures 4A–F, so *X* has a negligible role in its appearance or modulation. In contrast, the parameter *Y* encompasses corticothalamic gains, including *G*_*es*_, which most strongly modulates the alpha resonance, as seen in Figures 4E,F. It has also been previously established that theta resonance is enhanced as *Y* decreases through zero to negative values (Robinson et al., 2002), which is in accord with Figures 5A–D,G,H, and with a reduction in top-down gains.

The ERPs corresponding to these transfer functions reveal connections with the transfer functions' key characteristics. We observed in Figure 3B that the most obvious difference between the two evoked responses is the appearance of the late P200 feature in the response for the fixed-gain ERP parameter set, which is not present for the fixed-gain EEG set. Figures 4B,D,F confirm that cortical gains *G*_*ee*_, *G*_*ei*_, and *G*_*es*_, do not play significant roles in the generation of this feature. In fact, Figures 5B,D,H show that the appearance of the P200 feature is the consequence of decreasing the top-down gains, thereby paralleling the appearance of the theta peak in the transfer functions because an impulse response with the period of ≈ 250 ms is expected from a transfer function that shows a ≈ 4 Hz peak. Similarly, the early feature N100 is the direct consequence of the alpha peak ≈ 10 Hz in the transfer function resonances. Therefore, the prominent features of the ERP are related to key characteristics of the transfer functions, which are related to equivalent data filters (Babaie-Janvier and Robinson, 2018, 2019), as discussed in the next section.

### 3.3. Evoked Potentials With Dynamically Modulated Gains

In this section we aim to produce ERPs with features close to those generated by the fixed-gain ERP parameter set $T_{en}^{*}$ in Kerr et al. (2008) using the corticothalamic model with its gains, but starting at the fixed-gain EEG set with the gains being dynamically modulated as part of the response; i.e., *g*_*ab*_ ≠ 0. Henceforward we denote the transfer function with modulated gains and its corresponding time responses with superscript delta ($T_{en}^{\Delta }$ and $\phi_{e}^{\Delta }$). We keep the temporal parameters α, γ_*e*_, τ_*es*_, and τ_*se*_, fixed and we find the *g*_*ab*_ that is required to achieve $T_{en}^{*}$ so that the root mean square value of $T_{en}^{*}-T_{en}^{\circ }$ is minimized, using the parameter sensitivity results of section 3.2 as a guide for calculating local feedback strength *g*_*ab*_.

We first define a relative coefficient of modulation, denoted by Δ, which is the magnitude of the fractional change in $G_{ab}^{\left(ERP\right)}$ relative to $G_{ab}^{\left(0\right)}$

(39) $$\begin{array}{l} \Delta_{ab}=\frac{G_{ab}^{\left(ERP\right)}}{G_{ab}^{\left(0\right)}}-1, \end{array}$$

to investigate a variety of changes and establish a characteristic scale of such gain changes. Using the values of $G_{ab}^{\left(ERP\right)}$ and $G_{ab}^{\left(0\right)}$ in Table 1 we then compute Δ_*ab*_ in Equation (39) and determine the values of *g*_*ab*_ by requiring

(40) $$\begin{array}{l} G_{ab}^{\left(ERP\right)}=G_{ab}\left(s\right){|}_{s\to 0}, \end{array}$$

(41) $$\begin{array}{l} =G_{ab}^{\left(0\right)}+g_{ab}\phi_{b}^{\left(0\right)}, \end{array}$$

which yields the values of *g*_*ab*_ needed to produce results close to the fits to experimental ERPs by Kerr et al. (2011).

**Figure 6:**
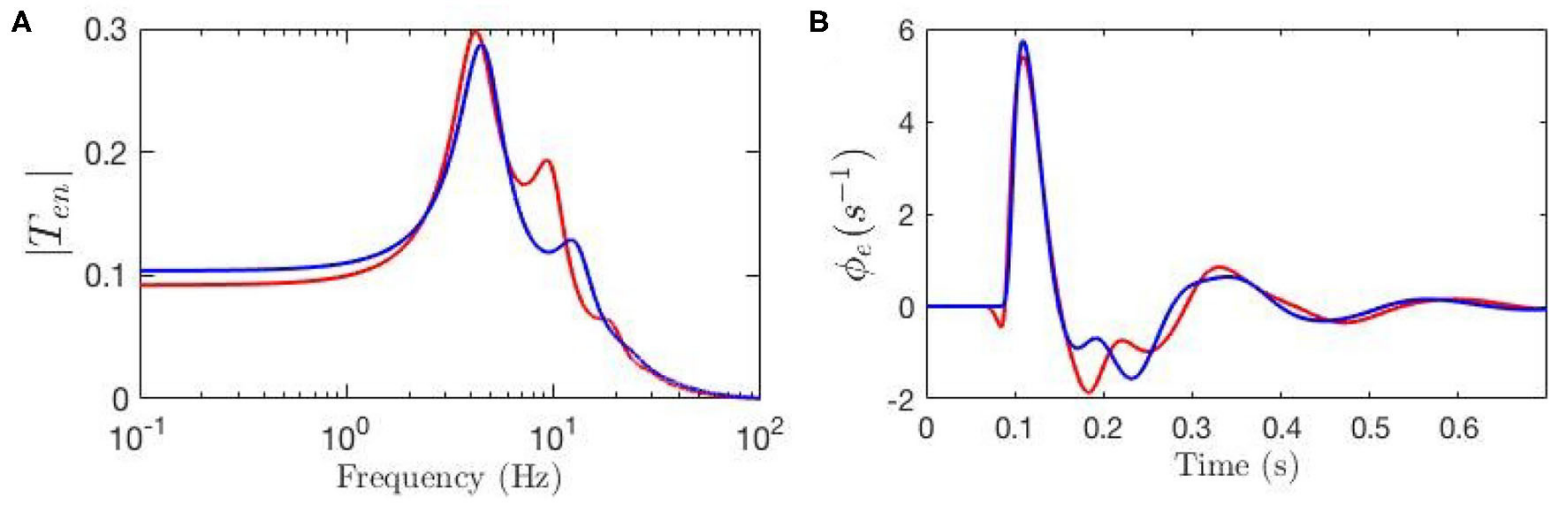
Transfer functions and corresponding ERPs for parameters fitted by Kerr et al. (2011), as listed in Table 1, and represented by blue line, and for modulated gain parameters represented by red line. **(A)** Magnitude of the transfer functions. **(B)** Model cortical ERPs.

We then examine the gain changes between experimental ERP fit and baseline to see which give rise to the key features of the ERP. This guides us in initializing Δ_*ab*_ and the relative magnitudes of the *g*_*ab*_ are then estimated by minimizing the rms deviation between theoretical and experimental ERPs over the time interval to post-stimulus, subject to the constraints that none of the *G*_*ab*_ can change sign, and neither can *X* and *Z*, because the excitatory or inhibitory nature of neural populations cannot change. This yields the *g*_*ab*_ shown in Table 2. Figures 6A,B compare the three transfer functions; i.e., experimental fit ERPs, baseline and modulated gain, and their generated ERPs. These results show that the modulated-gain transfer function and corresponding ERP represents the main features of the dynamics; i.e., early component N100 and late one P200, and it only exhibits slightly shifted theta, alpha, and beta peaks, while there is no P200 in the baseline. The ERP with modulated gain is accurate to an rms fractional error of 0.08. One general observation is that the gains are variously modulated during an ERP response to the stimulus; i.e., some increased substantially while others decreased, especially near the N100 peak.

**Table 2:** Numerically estimated values of modulation terms for normal adults in the alert, eyes-open state, with their effect on resulting gains based on Equation (39).

**Quantity**	**Description**	**Value**	**Effect (Δ_*ab*_) (%)**	**Unit**
**Gain parameters (dimensionless)**							
*g*_*ee*_	Feedback strength to *e* from *e*	−0.12	−30	−
*g*_*se*_	Feedback strength to *s* from *e*	−0.03	−20	−
*g*_*sr*_	Feedback strength to *s* from *r*	−0.05	+60	−
*g*_*es*_	Feedback strength to *e* from *s*	−0.03	−25	−
*g*_*re*_	Feedback strength to *r* from *e*	+0.06	+40	−
*g*_*ei*_	Feedback strength to *e* from *i*	−0.10	+20	−
*g*_*rs*_	Feedback strength to *r* from *s*	+0.001	≈ 0	−
*g*_*is*_	Feedback strength to *i* from *s*	−0.03	−25	−

*Plus and minus signs indicate increases and decreases in maximum magnitude, respectively*.

The effects of the estimated local feedback strengths *g*_*ab*_ on the overall dynamics of gain parameters were also calculated using Equation (15) and shown in Table 2. The results show that all inhibitory gains are significantly increased, with thalamic inhibitory gain *G*_*sr*_ doubled and cortical inhibitory gains increased by up to 60%. The other finding is that the intracortical excitatory gains *G*_*ee*_ and *G*_*ie*_ are reduced by one third during an ERP. Regarding corticothalamic gains, while the gain from cortex to LGN *G*_*se*_ shows a weakening, the gain to TRN *G*_*re*_ is strengthened up to 40% in magnitude, which in turn induces an indirect increase in the inhibitory activity of the thalamus *G*_*sre*_. Overall, all gains of the top-down feedback pathways that end in excitatory populations are weakened during ERP while the synapses to the inhibitory population have been strengthened.

**Figure 7:**
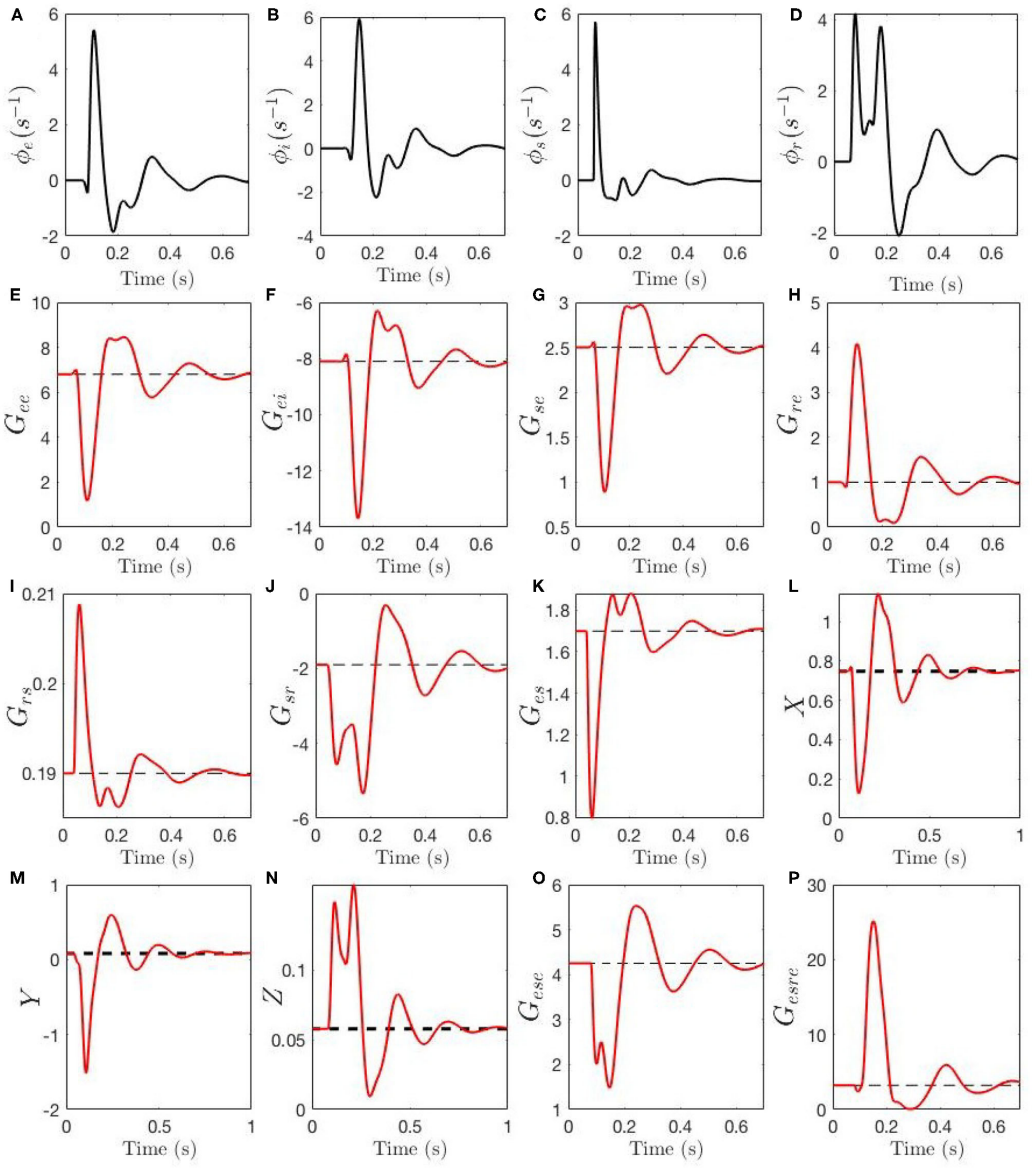
Temporal dynamics of corticothalamic gains during an ERP event. The steady-state $G_{ab}^{\left(0\right)}$ of each gain is shown by a black dashed line, the adjustment due to local feedback $G_{ab}^{\left(1\right)}$ around the steady-state and the total gain magnitude *G*_*ab*_ is shown by red line. Evoked time evolution of the following quantities are shown: **(A)** ϕ_*e*_. **(B)** ϕ_*i*_. **(C)** ϕ_*s*_. **(D)** ϕ_*r*_. **(E)** *G*_*ee*_. **(F)** *G*_*ei*_. **(G)** *G*_*se*_. **(H)** *G*_*re*_. **(I)** *G*_*rs*_. **(J)** *G*_*sr*_. **(K)** *G*_*es*_. **(L)** *X*. **(M)** *Y* **(N)** *X*. **(O)** *G*_*ese*_. **(P)** *G*_*esre*_.

Figure 7 shows the evolution of the activities ϕ_*a*_, gains *G*_*ab*_, compound gains *G*_*ese*_, *G*_*esre*_, and loop feedback parameters *X*, *Y*, and *Z* during an ERP. In Figures 7A,B we see that ϕ_*e*_ and ϕ_*i*_ have closely synchronized evolution, with a sharp peak around 100 ms, followed by decaying theta-band oscillations. Figure 7C shows that ϕ_*s*_ has similar evolution, but with an even sharper peak. We see the reason for this peak being truncated in Figure 7D, where the sudden initial rise in ϕ_*r*_ causes suppression of ϕ_*s*_ via the gain *G*_*sr*_. The drop in ϕ_*s*_ then reduces excitation of ϕ_*r*_ around 100 ms, followed by a second peak then decay.

Figures 7E–K show the evolution of the gains. The intracortical gains *G*_*ee*_ and *G*_*ei*_ evolve in phase, with *G*_*ee*_ decreasing sharply around 100 ms, while *G*_*ei*_ (a negative gain) increases in magnitude; both display subsequent nearly synchronized decaying theta oscillations. The corticothalamic gains *G*_*re*_ and *G*_*se*_ display antiphase dynamics, with coupling to the reticular nucleus increasing, and that to the relay nuclei decreasing, around 100 ms. Intrathalamic delays *G*_*sr*_ and *G*_*rs*_ have evolution that closely mirrors the evolution of their drivers ϕ_*r*_ and ϕ_*s*_: the gain *G*_*rs*_ for the effect of *s* on *r* rises sharply early in the ERP, but only by a small amount, whereas *G*_*sr*_ increases by a large factor due to the large relative increase in ϕ_*r*_. Finally, the thalamocortical gain *G*_*es*_ has an evolution that mirrors that of *G*_*rs*_ due to their common drive, but which is inverted because of the relative signs of *g*_*rs*_ and *g*_*es*_.

The effects of the above dynamics are summarized in Figures 7L–N. We see that the intracortical feedback parameter *X* initially decreases sharply, owing to the simultaneous decrease of *G*_*ee*_ and increase of |*G*_*ei*_|. Subsequently, it undergoes damped oscillations. The corticothalamic loop parameter *Y* changes sign from slightly positive to strongly negative, which suppresses alpha activity and enhances theta, before also oscillating. The intrathalamic parameter *Z* increases by a significant factor, but remains too small for spindle-frequency oscillations to become prominent. Further light is cast on the dynamics of *Y* by considering the two opposing contributions *G*_*ese*_ and *G*_*esre*_ in Equation (32), shown in Figures 7O,P. The excitatory corticothalamic gain *G*_*ese*_ regulates alpha activity and inhibitory corticothalamic gain *G*_*esre*_ enhances theta activity, and therefore, decreased *G*_*ese*_ along with increased *G*_*esre*_ cause *Y* to decrease (see section 2.3), which results in the appearance of a theta resonance in parallel with alpha suppression.

### 3.4. ERP Corticothalamic Filters

**Figure 8:**
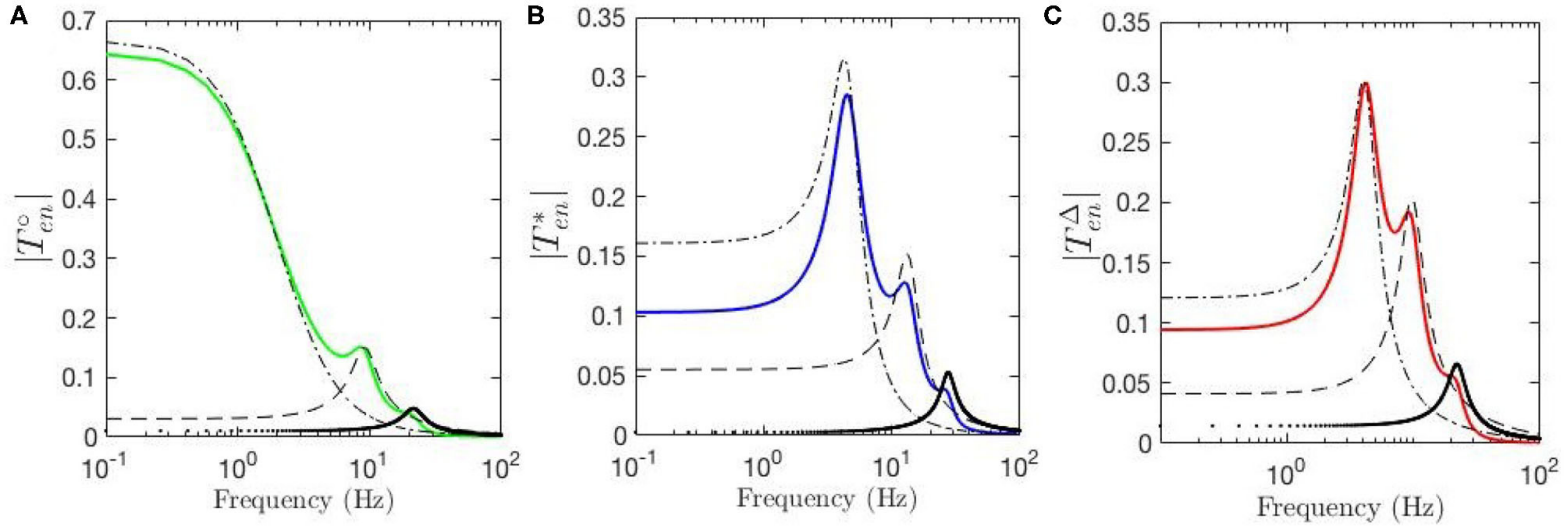
Magnitudes of the transfer functions and their retrieved filters vs. frequency. Here, ℓ represents the low-frequency filter without oscillations, the dash-dot lines represent theta filters, the dashed lines represent alpha filters, and the dotted lines represent beta filters. **(A)** $T_{en}^{\circ }$ for fixed-gain resting EEG parameters. **(B)** $T_{en}^{*}$ for fixed-gain ERP parameters of Kerr et al. (2008). **(C)** $T_{en}^{\Delta }$ for the gain-modulated model with initial resting EEG parameters.

Temporal aspects of the ERPs, such as the appearance of early and late features N100 and P200 and their magnitudes, were studied in the previous sections. Our approach enables us study these oscillatory properties of the ERPs using the control-systems approach of filter identification from the corresponding transfer functions. Six-pole approximations for $T_{en}^{\Delta },\;T_{en}^{\circ }$, and $T_{en}^{*}$ are made using the relevant equations in section 2.4. Figure 8A shows the resulting transfer function for fixed-gain resting EEG parameters. We see that $T_{en}^{\circ }$ can be decomposed into three filter responses that emphasize low, alpha, and beta frequency ranges, respectively. The low-frequency filter, denoted by ℓ, exhibits a resonance at 0 Hz; the alpha filter, denoted by A, has a resonance at about 9.5 Hz; and the beta filter, denoted by B, has a resonance at about 18 Hz. Figure 8B shows the filters obtained for the transfer function using static-gain ERP parameters from Kerr et al. (2008) $T_{en}^{*}$, in which both beta and alpha filters are present with magnitudes and peaks slightly different from $T_{en}^{\circ }$. However, the third filter obtained for low frequency regime shows a resonance in the theta range, unlike the result for the fixed-gain resting EEG transfer function, which we denote by T. The data filters derived for the modulated-gain transfer function $T_{en}^{\Delta }$, shown in Figure 8C, are similar to the one for the fixed-gain ERP parameters, $T_{en}^{*}$, as expected. As we saw in section 3.3 the appearance of the theta resonance is associated with a decrease in *Y* which is the result of the decrease in top-down corticothalamic projections. Closer analysis shows that, as one moves from the fixed-gain EEG parameters to the fixed-gain ERP set, two zero-frequency poles collide and emerge with nonzero oscillation frequency in the *s*-plane, thereby yielding theta oscillations in the ERP. The alpha and beta filters each comprises a complex conjugate pair of poles, resulting in an oscillation in the temporal response at the corresponding frequency. The low-frequency filters obtained for both $T_{en}^{\circ }$ and $T_{en}^{*}$ exhibit a peak in the theta frequency range (3 − 7 Hz). This is a key difference between the dynamic-gain ERP response and static-gain response in which the slow filter is an underdamped filter that has its two poles on the real axis. In other words, the poles of the slow filter become complex conjugates that generate a theta response in the dynamic-gain transfer function, whereas they are purely damped poles in the static-gain case. This is consistent with standard ERP observed in experiments, both in auditory and visual modalities.

**Figure 9:**
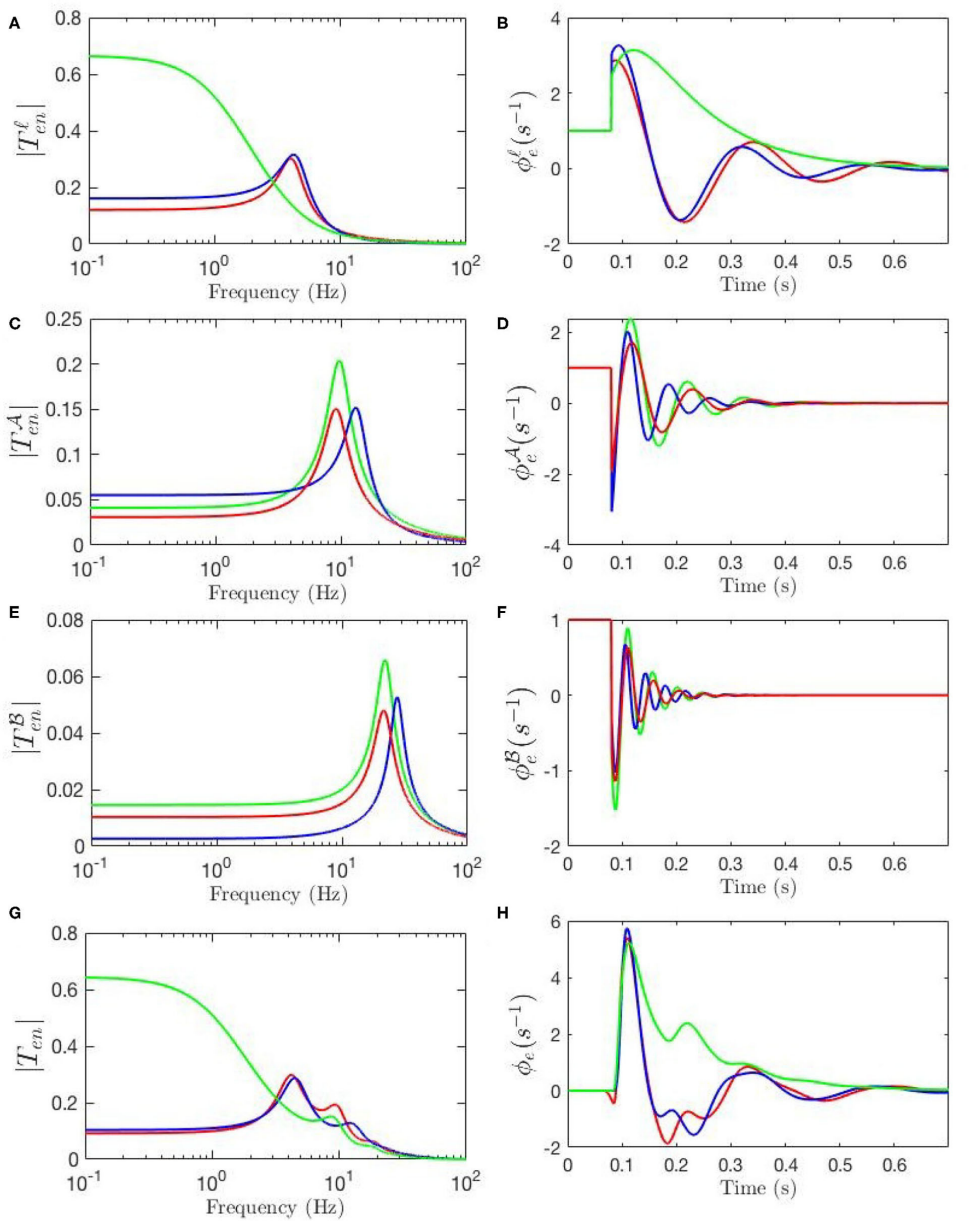
Magnitudes of the transfer functions, their retrieved filters, and corresponding ERPs. Green lines denote fixed-gain resting EEG parameters, blue lines denote fixed-gain ERP from Kerr et al. (2011), and red lines show modulated gain parameters. **(A)** Magnitudes of low-frequency filters vs. frequency. **(B)** ERP Partial responses generated by low-frequency filters. **(C)** Magnitudes of alpha filters vs. frequency. **(D)** ERP partial responses generated by alpha filters. **(E)** Magnitudes of beta filters vs. frequency. **(F)** ERP partial responses generated by beta filters. **(G)** Magnitudes of full transfer functions vs. frequency. **(H)** ERP responses generated by full transfer functions.

Our model also allows determination of the partial temporal responses corresponding to each filter where the total evoked potential is obtained by summing these responses. This is particularly valuable for studying the direct relationship between the temporal features of the ERPs and their oscillatory properties where the data filters' roles in generating them can be revealed. Figure 9G compares the three above-mentioned transfer functions in frequency domain and Figure 9H compares their corresponding evoked potentials in time domain. The key difference centers on the lack of the theta resonance, and thus the P200 feature, in the response starting from background EEG parameters.

Figure 9A compares the low frequency regime filters obtained for the three transfer functions and Figure 9B compares their contributions in the ERP response, which shows that the late feature P200 is present in the temporal response of the ERP filter while it is not present in the response generated by the slow filter of the resting-EEG transfer function. This confirms the role of theta filter in generating P200. Figures 9C,E show the alpha and beta filters obtained for the transfer functions, and Figures 9D,F show their contribution in the evoked potentials, which show all three transfer functions have very similar alpha and beta filters. The N100 feature is present in all three partial responses which shows that early feature is governed by these filters.

### 3.5. Evoked Potentials and Attentional Modulation

Evoked potentials are widely used to study attention, particularly those aspects not observable with behavioral methods (for a review see Woodman, 2010). It has been shown that attention modulates the key features in the response (Hillyard and Anllo-Vento, 1998; Hillyard et al., 1998; Herrmann, 2001; Herrmann and Knight, 2001); however, the exact mechanisms underlying such changes are unknown. Here we have reproduced evoked potentials by using modulated gain transfer function that starts by EEG baseline parameters and dynamically adjust the gains during the process so that its response contains key features very similar to those of experimental ERPs. This has enabled us to investigate the effects of attentional modulation on activities in the corticothalamic system. We have showed that both early and late features of evoked potentials are modulated by local feedbacks that dynamically adjust the gains to respond to stimulus. We know from experiments that such modulations are related to attention (Luck et al., 1997, 2000; Herrmann and Knight, 2001). Babaie-Janvier and Robinson (2019) showed that parameters of gains can dynamically adjust to enable estimation of incoming signals via a form of attention, in which dynamic gain changes increase the weight attached to stimulus rate of change when sudden changes occur, and to stimulus value under static conditions.

**Figure 10:**
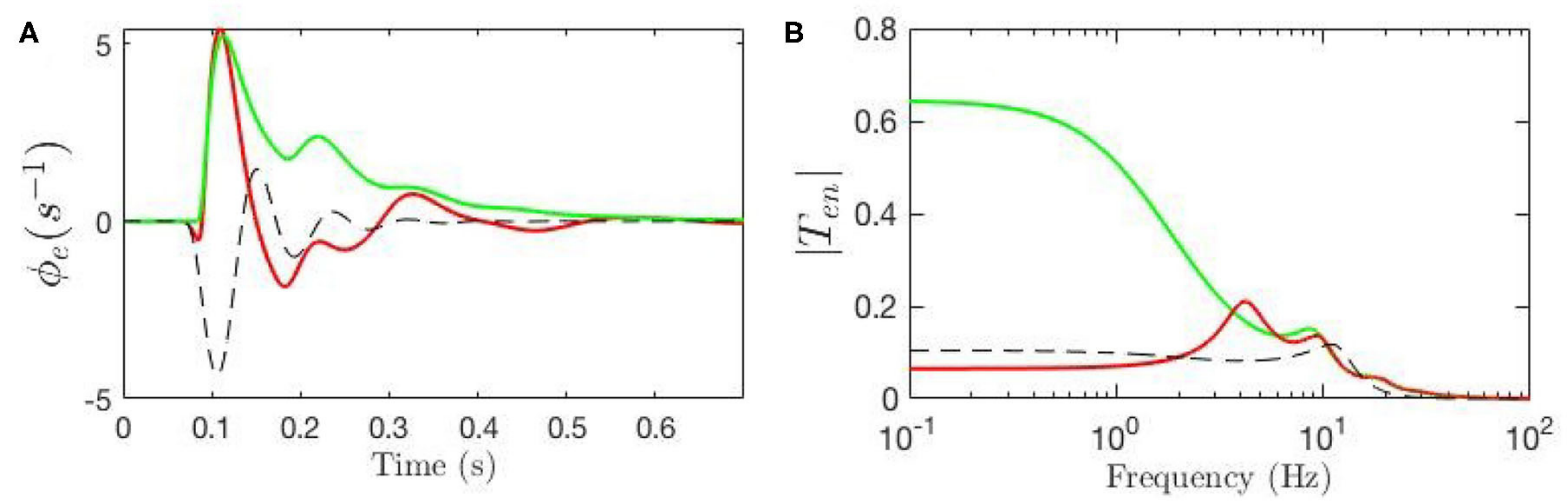
Evoked potentials for the static-gain EEG parameters (green curve) and modulated-gain parameters starting from the same initial values (red). The dashed black line shows the contribution of the gain modulation, which adds to the green curve to give the red one. **(A)** Model cortical ERPs **(B)** Magnitude of the transfer functions.

Our model allows us to estimate the parts of the ERP that are explicitly induced by gain changes and interpret them in terms of attention. Figure 10A shows the baseline ERP in green, while the contribution made by gain modulation is shown by the dashed black line; the sum of these two contributions gives the total ERP, shown in red. We also calculated the magnitude of the transfer function responsible for the contribution which is shown in Figure 10B. The dominant contribution has a frequency of about 12.5 Hz. This contribution is sizeable from about 50 − 250 ms with maximal amplitude around 150 ms.

The levels of oscillatory activity in human cortex are also observed to be altered by attention (Hillyard and Anllo-Vento, 1998; Hillyard et al., 1998; Herrmann, 2001; Yamagishi et al., 2003; Sauseng et al., 2005; Thut et al., 2006; Klimesch et al., 2007; Wang, 2010). Our model relates the mechanism by which these oscillations are produced to the attentional impact they have on evoked potentials through gain dynamics. This is particularly useful because it allows us further investigate the role of various gains in attention as well as to locate the connections and structures from which these impacts originate. Based on the results shown above, the early features are mainly governed by alpha and beta filters, which is in accord the experimental findings (Hillyard and Anllo-Vento, 1998; Hillyard et al., 1998). One significant finding is that we observe a suppression of alpha activity and a slight increase in beta activity, both in accord with the literature on alpha suppression during attentional tasks, dating back to the pioneering work of Berger (Berger, 1929a,b; Marrufo et al., 2001; Ward, 2003; Sauseng et al., 2005). In the corticotahalamic model, the excitatory loop gain *G*_*ese*_ principally governs the alpha activity, which comprises two gains *G*_*es*_ and *G*_*se*_, both are shown to decrease significantly in the early phases of the response, as shown in Figures 7F,K. This explains why the transfer function of baseline EEG shows a dominant alpha peak and ERP responses exhibit suppressed alpha activity (see Figures 9C–F). The low-frequency filter has little impact on this part of the response, whereas its theta frequencies are shown to dominate the later phases of the response, where attentional modulation has found to be most significant (Hillyard and Anllo-Vento, 1998; Hillyard et al., 1998; Herrmann, 2001).

## 4. Summary and Discussion

We have modeled event related potentials by using neural field theory (NFT) of the corticothalamic system to incorporate attentional gain modulation via local synaptic feedbacks. Our model provides a unified explanation of the resting EEG and standard ERPs. Furthermore, the model uses a dynamic gain modulation scheme to explain the ERP and interpret part of it as being due to attention. The main findings are:

- We have calculated standard ERPs using an extended NFT model of large-scale activity in the corticothalamic system, which embodies local feedbacks that modulate the gains of neural activity as part of the response to incoming stimuli. First, we treated all the parameters as static and predicted the ERP using both a prior set of EEG resting-state parameters and a set of static gain parameters fitted to standard ERP experiments by Kerr et al. (2008). It was shown that there is a significant contrast between the dynamics of the ERP responses as well as between their underlying transfer functions. While the ERP response produced using experimentally-fitted parameters by Kerr et al. (2008) shows a typical response in both early and later phases, the ERP generated by using background-EEG parameters is dominated by a sudden rise followed by a slow decay, quite different from experiment.
- Transfer functions for both sets of static-gain parameters were calculated and their corresponding data filters were obtained, which are associated with the basic modes responsible for low-frequency, alpha, and beta responses. It was shown that alpha and beta resonances were present in both resting EEG and ERP responses; in contrast, the ERP low-frequency regime (< 5 Hz) contains a theta resonance, whereas this filter has only a zero-frequency resonance for background-EEG parameters.
- A sensitivity analysis showed that top-down connections and cortical excitatory connections must be considerably weakened during an ERP event to produce a theta resonance. This result agrees with a previous hypothesis that the importance of the internal model (top-down signals) is reduced in parallel with enhanced emphasis on external information, with the result that the brain attends to new stimuli and suppresses prior mean signal levels as they become less salient (Friston, 2010; Babaie-Janvier and Robinson, 2019). Changes in loop-gain parameters, *X*, *Y*, and *Z* caused by gain changes also support the trade off between introspection and external attention. The decrease in top-down signals corresponds to a reduction in the corticothalamic loop gain parameter *Y* as this loop's positive feedback decreases.
- Inclusion of dynamic gain modulation, informed by the analysis in (iii), enabled experimental ERPs to be fitted, starting from background EEG parameters.
- We studied both the static-gain and modulated-gain transfer functions via control theory in terms of system resonances that were recently shown by Babaie-Janvier and Robinson (2019) to implement data filtering whose gain adjustments can be interpreted as attention. The onset of a sudden change dynamically enhances the theta filter and de-emphasizes the alpha and beta filters, which can be interpreted as attentional suppression of alpha and beta oscillations. This is in accord with the literature on alpha suppression during attentional tasks dating back to the pioneering work of Berger (Berger, 1929a,b; Marrufo et al., 2001; Ward, 2003; Sauseng et al., 2005; Klimesch et al., 2007). The results show that resonant filters deriving the low-frequency oscillations in an evoked potential response have different instantaneous parameters than those responsible for low-frequency resting EEG responses (which results in strong theta oscillations in the ERP response whereas resting EEG lacks them), while both responses share similar alpha and beta resonant filters. In this framework, the very low frequency response emphasizes mean values, while alpha and beta responses seen at around 80 − 200 and 180 − 280 ms, respectively, reflect fast changes, as does the theta response at around 150-400 ms; and overall timescale gates rate at which new stimuli can be processed. Such attentional adjustments are in accord with literature (Hillyard and Anllo-Vento, 1998; Hillyard et al., 1998; Herrmann, 2001; Herrmann and Knight, 2001) and we showed that both early and late features of ERPs involve such gain modulations, which dynamically increase the weight attached to stimulus rate of change when sudden changes occur, and to stimulus value under static conditions (Babaie-Janvier and Robinson, 2019).

Overall, our model enables both activity changes and gain (i.e., effective connectivity) changes to be calculated as parts of a generalized evoked response, which allowed us estimate the part of ERP response that is explicitly induced by gain modulations and interpret it in terms of attention in response to sudden changes. Notably, instead of postulating a starting state for ERPs with parameters different from those of background EEG, as done by Kerr et al. (2008), we have showed that the same starting point can yield a similarly good match with data when dynamically modulated gains are included. These outcomes also enable further investigation of the roles of various biophysical process that may be involved in such gain adjustment in the brain during attention.

## Data Availability Statement

The original contributions presented in the study are included in the article/supplementary material, further inquiries can be directed to the corresponding author/s.

## Author Contributions

TB-J and PR conceived the research, developed the theory, verified the analytical methods, and discussed all the results. TB-J performed the computations, worked out the technical details, performed numerical calculations, produced figures and numerical results, and drafted the first draft of the manuscript. All authors contributed to the final manuscript.

## Conflict of Interest

The authors declare that the research was conducted in the absence of any commercial or financial relationships that could be construed as a potential conflict of interest.
